# Population Screening for Diabetes and Prediabetes in North Macedonia and Bosnia and Herzegovina: A Transnational Epidemiological Report

**DOI:** 10.7759/cureus.100726

**Published:** 2026-01-04

**Authors:** Tatjana Milenkovic, Zelija Velija Asimi, Ivica Smokovski, Nadica Bozhinovska Dimova, Azra Burekovic

**Affiliations:** 1 Endocrinology, Diabetes and Metabolism, University Clinic of Endocrinology, Diabetes and Metabolic Diseases, Faculty of Medical Sciences, University St. Cyril and Methodius, Skopje, MKD; 2 Endocrinology and Diabetes, SSST University (Sarajevo School of Science and Technology University), Sarajevo, BIH; 3 Endocrinology and Diabetes, SSST University (Sarajevo School of Science and Technology University) Polyclinic UniMed Sarajevo, Sarajevo, BIH; 4 Faculty of Medical Sciences, University Goce Delcev, Stip, MKD; 5 Endocrinology, University Clinic of Endocrinology, Diabetes and Metabolic Disorders, Skopje, MKD; 6 Endocrinology, Diabetes and Metabolism, Clinical Hospital Acibadem Sistina, Skopje, MKD; 7 Endocrinology, Diabetes and Metabolism, University Clinical Hospital for Endocrinology, Diabetes and Metabolic Diseases, Sarajevo, BIH

**Keywords:** health system research, pre-diabetes, prevalence of diabetes, screening, type 2 diabetes mellitus

## Abstract

While North Macedonia (NM) and Bosnia and Herzegovina (BH) have a relatively high prevalence of diabetes, both countries have a low diagnostic rate due to the lack of unified eHealth systems.

We conducted a small-scale, general screening epidemiologic study in NM and BH for a better understanding of the high prevalence of diabetes in our region. A total of 1,291 individuals at risk of diabetes were studied. The diagnostic criteria for diabetes included fasting plasma glucose (FPG) ≥7.0 mmol/L or glycated hemoglobin (HbA1c) ≥6.5%, while prediabetes was diagnosed using impaired glucose tolerance (IGT) or impaired fasting glucose (IFG). The prevalence of diabetes and prediabetes was higher in NM, with a combined rate of 78% compared to 71% in BH. After adjusting for gender, smoking, waist circumference, and triglycerides, the NM group is 1.45 times at greater risk for diabetes. General population screening for diabetes and prediabetes can serve as a cost-effective approach to address the escalating diabetes burden in the Western Balkans. Collaborative initiatives to enhance the integration of national health systems, ensure data reliability, and prioritize preventive healthcare are essential for achieving sustainable diabetes management.

## Introduction

North Macedonia (NM) and Bosnia and Herzegovina (BH), located in South-Eastern Europe, have the second-highest prevalence of diabetes in Europe, primarily due to type 2 diabetes (T2DM) [1,2]. The estimated diabetic population (20-79 years) is 366,900 in BH and 188,800 in NM [3,4]. Contributing factors include shared dietary habits, sedentary lifestyles, high unemployment during economic transitions, and high smoking rates. The total estimated diabetes prevalence in these countries, 7.4% in NM and 12.4% in BH, poses a significant socio-economic burden, necessitating cost-effective healthcare strategies based on International Diabetes Federation (IDF) data [3]. In NM, national eHealth systems introduced in 2013 have improved diabetes monitoring, though the diagnostic rate remains low, with diagnosed prevalence at 5.0% versus the IDF-estimated 7.4%. In BH, the lack of a unified health IT system contributes to underreported diabetes cases, with estimates ranging from 4% to 6% versus the IDF-estimated 12.2% [2-6]. Therefore, to better characterize the diabetes epidemic in our region, we conducted a population-based pilot epidemiological study to assess the prevalence of diabetes and prediabetes.

General screening

Screening for diabetes and prediabetes in the general population aims to identify individuals at risk of developing type 2 diabetes mellitus (T2DM) and associated complications, including cardiovascular and kidney diseases. Early detection facilitates timely interventions to prevent disease progression and reduce long-term health impacts [7-9]. We conducted a pilot epidemiological screening study in NM and BH for a better understanding of the high prevalence of diabetes in our region.

## Materials and methods

A total of 1,291 individuals at risk of diabetes were studied across NM (342) and BH (949). Diagnostic criteria for diabetes included FPG ≥7.0 mmol/L or HbA1c ≥6.5%, while prediabetes was diagnosed using impaired glucose tolerance (IGT) or impaired fasting glucose (IFG). Diabetes and pre-diabetes cases were stratified in the following age groups: below 20 years, 20-39 years, 40-59 years, 60-79 years, 80 years or older; and further sub-stratified by gender and place of residence (urban/rural). Data were collected at the time of testing include age, gender, outcome (normoglycemia, pre-diabetes, diabetes), date of test, FPG, 2-hour plasma glucose following 75 g oral glucose load, HbA1c, body weight and body height, rural or urban place of residence, smoking status, waist circumference, systolic and diastolic blood pressure, total cholesterol, low-density lipoprotein (LDL) cholesterol, high-density lipoprotein (HDL) cholesterol, triglycerides, creatinine. The doctors from different countries collaborated by collecting data (on the previously arranged conditions and criteria as mentioned above) within primary care clinic settings, and all laboratory analyses conducted as part of the study utilized standardized assays. Afterwards, the collected data was scanned and sent through email for data interpretation and statistics. The data were collected in the time period from 2021 to 2022.

Ethics statement

This study was conducted in accordance with the principles of the Declaration of Helsinki. The protocol was approved by the University Clinic of Endocrinology, Diabetes and Metabolism in Skopje, North Macedonia, and the Medical Faculty of the University of Sarajevo in Bosnia and Herzegovina. Written informed consent was obtained from all participants.

## Results

In the NM group, there were more men than women, in contrast to the BH group, where there were more women than men. The average age of NM subjects was 55.14±13.22, and the average age of BH subjects was 53.40±14.99 years. Regarding age, there was a similar distribution by strata. More urban residents were in the NM group as compared to the BH group. The number of smokers was similar. The total number of people with T2DM and prediabetes in the NM group is higher than in the BH group, 78% vs 71%.

Both examined groups were overweight (28.73±4.65 vs 29.51±5.43 kg/m2). Fasting glucose values were higher in the NM group, while lipid and blood pressure values were significantly higher in the BH group (Table 1).

**Table 1:** Characteristics of the study population Values were expressed as means ± (SD) and absolute numbers (%) depending on the type of data. Group means were compared with Student’s t-tests. Categorical variables were analysed by the chi-square test. P value=statistical significance; * statistically significant Age expressed in years; Rural residence - living outside the city; Urban residence - living in the city; Weight expressed in kilograms. Height expressed in centimeters. Waist expressed in centimeters. BMI=body mass index (kg/m2); FPG=fasting plasma glucose (mmol/L); 2h glucose (mmol/L); total cholesterol (mmol/L); LDL=low density lipoprotein (mmol/L); HDL=high density lipoprotein (mmol/L); SBP=systolic blood pressure (mmHg); DBP=diastolic blood pressure (mmHg), creatinine (umol/L)

		North Macedonia (n=342)	Bosnia and Herzegovina (n=949)	p-value
Estimated diabetes prevalence		7.4%	12.2%	
Gender	Male	181(52.9%)	352(37.1%)	<0.001 *
	Female	161(47.1%)	597(62.9%)	
Age group	<40	38(11.1%)	191(20.1%)	0.004 *
	40~49	76(22.2%)	171(18.0%)	
	50~59	94(27.5%)	235(24.8%)	
	60~69	78(22.8%)	219(23.1%)	
	70~	56(16.4%)	133(14.0%)	
Residence	Rural	67(19.9%)	255(28.5%)	0.002 *
	Urban	270(80.1%)	641(71.5%)	
Smoking	Non-smoker	233(68.9%)	592(65.7%)	0.283
	Smoker	105(31.1%)	309(34.3%)	
Examination outcome	Normoglycemic	76(22.2%)	277(29.2%)	0.003 *
	Prediabetes	115(33.6%)	345(36.4%)	
	Diabetes	151(44.2%)	327(34.5%)	
Age		55.14±13.22	53.40±14.99	0.045 *
Weight		82.35±14.88	86.88±16.82	<0.001 *
Height		169.31±9.00	171.64±8.80	<0.001 *
Waist		97.72±17.35	97.62±14.71	0.917
BMI		28.73±4.65	29.51±5.43	0.012 *
FPG		7.08±2.55	6.65±2.31	0.007 *
2h glucose		8.04±3.33	8.23±2.95	0.410
HbA1c		6.46±1.73	6.50±1.54	0.755
Total cholesterol		5.18±1.15	5.70±1.51	<0.001 *
LDL cholesterol		3.14±1.19	3.36±1.04	0.009 *
HDL cholesterol		1.46±0.70	1.48±0.78	0.719
Triglycerides		2.02±1.37	2.26±1.64	0.019 *
SBP		130.24±17.12	134.54±17.69	<0.001 *
DBP		81.73±8.63	82.90±10.45	0.044 *
Creatinine		75.90±16.39	83.10±19.92	<0.001 *

In this study, demographic differences were observed between the two groups: NM had a higher proportion of men (52.9%), while BH had a higher proportion of women (62.9%). The average age was similar, with 55.1 years in NM and 53.4 years in BH. The prevalence of diabetes and prediabetes was higher in NM, with a combined rate of 78% compared to 71% in BH. Both groups were classified as overweight; however, NM participants exhibited higher fasting plasma glucose (FPG) levels, while BH participants displayed worse lipid and blood pressure profiles. After adjusting for gender, smoking, waist, and triglycerides, the NM group is 1.45 times at greater risk for diabetes when compared to the BH group. Also, urban living increases the risk of diabetes by 1.69 times (Table 2).

**Table 2:** Diabetes risk adjusted for gender, smoking, waist (centimeters), and triglycerides (mmol/L) Urban - living in the city; Age expressed in years; BMI - Body mass index (kg/m^2^); TC - total cholesterol (mmol/L); SBP - Systolic blood pressure (mmHg)

	B	p	RR(95% C.I.)
Macedonia	0.370	0.016	1.45(1.07~1.95)
Urban	0.527	0.001	1.69(1.23~2.33)
Age	0.046	<0.001	-
BMI	0.068	<0.001	-
TC	0.110	0.025	-
SBP	0.011	0.010	-

## Discussion

North Macedonia and Bosnia and Herzegovina, as countries on the Balkan Peninsula, have been experiencing notably high prevalence rates of type 2 diabetes compared to many other regions in Europe [3]. Several contributing factors include rising rates of obesity, sedentary lifestyles, unhealthy dietary habits, and genetic predisposition among the populations. Economic transitions and urbanization in Balkan countries have led to changes in traditional diets, often increasing the consumption of processed foods and sugars, while stimulating reduced physical activity. Additionally, limited access to preventive healthcare and public health education in certain areas exacerbates the problem [2]. According to the International Diabetes Federation (IDF), the countries in the Balkan region, such as Bosnia and Herzegovina and North Macedonia, have among the highest prevalence rates for type 2 diabetes in Europe [3]. Lifestyle factors, particularly diet and inactivity, play significant roles in diabetes prevalence. The progression from prediabetes to T2DM is substantial, with targeted interventions, including lifestyle modifications and pharmacotherapy, proving effective [10]. The IDF Diabetes Atlas highlights regional disparities, emphasizing the need for targeted prevention strategies [3]. However, barriers such as low awareness, cultural factors, and implementation challenges limit widespread adoption of screening tools [11-13]. Implementing awareness campaigns tailored to local contexts can help educate the public about diabetes risk factors, symptoms, and the importance of early detection. Collaboration with community leaders and healthcare providers ensures that messages reach a broad audience. This study, though limited in scale, holds significant value for advancing our understanding of the exceptionally high prevalence of diabetes within our region. Its findings offer essential insights into the factors contributing to this widespread health concern. Notably, this is the first study of its kind to be conducted in this area. As a pioneering effort, it lays the groundwork for the development of a unified and effective strategy aimed at reducing the risk of diabetes among the local population. The knowledge gained from this research is expected to guide future initiatives and inform public health interventions targeted at minimizing diabetes risk in our community.

## Conclusions

The study was pioneering, highlighting the greater need for population screening, especially in higher-risk population profiles: urban, overweight, sedentary, over 50 years of age, with higher blood pressure and lipid levels. It can also be inferred that telehealth support and guidance can facilitate early diagnosis and encourage better public health policies and more preventive interventions to prevent the development of diabetes.
